# Profils épidémiologique, clinique et virologique de la grippe en République démocratique du Congo de 2009 à 2018

**DOI:** 10.11604/pamj.2025.50.68.33798

**Published:** 2025-03-10

**Authors:** Saleh Muhemedi, Paul Lusamba, Edith Nkwembe, Léopold Lubula, Léonie Manya, Pélagie Babakazo

**Affiliations:** 1 Ecole de Santé Publique de Kinshasa, Université de Kinshasa, Kinshasa, République démocratique du Congo; 2 Institut National des Recherches Biomédicales, Ministère de la Santé, Hygiène et Prévention, Kinshasa, République démocratique du Congo; 3 Direction de Surveillance Epidémiologique, Ministère de la Santé, Hygiène et Prévention, Kinshasa, République démocratique du Congo

**Keywords:** Surveillance sentinelle, grippe, République démocratique du Congo, Sentinel surveillance, influenza, Democratic Republic of Congo

## Abstract

**Introduction:**

depuis 2007, la République démocratique du Congo a mis en place un système de surveillance sentinelle de la grippe qui a fonctionné avec 11 formations sanitaires. Ces établissements de soins prélevaient des échantillons oro et naso- pharyngés auprès des patients suspects de la grippe en utilisant les définitions des cas. Ces échantillons ont ensuite été envoyés au Laboratoire National de la Grippe pour analyse. Cette étude décrit les profils épidémiologique, clinique et virologique de ces patients.

**Méthodes:**

il s'agit d'une étude transversale, basée sur une revue documentaire effectuée à partir de fiches de notification de cas suspects de grippe, allant de janvier 2009 à décembre 2018. Les variables telles que le sexe, l'âge, les symptômes, le site sentinelle de provenance, la catégorie du patient, le type et sous type viraux identifiés et la période de survenue des cas, ont été exploités.

**Résultats:**

sur un total de 18461 cas notifiés, 1795 ont été positifs au virus influenza soit 9,7%; les patients de moins de 5 ans représentaient 53,1% des cas positifs; le virus du type A était présent dans 68% de cas contre 31,5% pour le type B; les cas positifs SARI représentaient 21% vs 79% pour les cas positifs ILI; la quasi-majorité des cas étaient survenus pendant la saison pluvieuse.

**Conclusion:**

cette étude nous a permis de déterminer l'ampleur de la grippe parmi les infections respiratoires aiguës et la fréquence des virus grippaux en circulation.

## Abstract (en)

**Introduction:**

since 2007, the Democratic Republic of the Congo has implemented a sentinel influenza surveillance systems, operating across 11 healthcare facilities. These facilities collected oropharyngeal and nasopharyngeal samples from patients suspected of having influenza, based on case definitions. The collected samples were then sent to the national influenza laboratory at the INRB for analysis. This study describes the epidemiological, clinical, and virological profiles of these patients.

**Methods:**

we conducted a cross-sectional study based on a documentary review of notification forms of suspected influenza cases, from January 2009 to December 2018. The variables analyzed included sex, age, symptoms, sentinel site, patient category, identified viral type and subtype, and the period occurrence.

**Results:**

out of a total of 18,461 reported cases, 1,795 (9.7%) tested positive for the influenza virus. Children under five years old accounted for 53.1% of positive cases. Influenza type A was present in 68% of cases, while type B accounted for 31.5%. Positive SARI (Severe Acute Respiratory Infection) cases represented 21%, whereas positive ILI (Influenza-Like Illness) cases accounted for 79%. The vast majority of cases occurred during the rainy season.

**Conclusion:**

this study enabled us to determine the burden of influenza among acute respiratory infections and the prevalence of circulating influenza viruses.

## Introduction

Faisant suite à l'émergence en 1997, d'un virus aviaire hautement pathogène A/H5N1, en Asie et qui s'est propagé en Europe en 2005 et en Afrique en 2006 (Egypte, Nigeria, Cameroun...), infectant aussi bien la volaille que l'homme [1], certains pays d'Afrique ont mis en place des systèmes de surveillance de la grippe avec l'appui de l'OMS et du CDC/Atlanta [2]. C'est par exemple le cas du Kenya [3], de la Tanzanie [4], du Rwanda [5], du Nigeria [6], du Sénégal [7] et de la Zambie [8]. Ces pays étaient parmi les premiers à partager leurs expériences en matière de surveillance de la grippe. Pour la République démocratique du Congo, c'est en 2007 que le système de surveillance sentinelle de la grippe a été mis en place avec comme objectifs: (i) détecter de nouvelles souches grippales capables de causer une pandémie; (ii) déterminer l'épidémiologie de la grippe et d'autres maladies respiratoires virales; (iii) suivre les tendances en ce qui concerne le nombre de malades de décès imputables aux syndromes respiratoires aiguës sévères (SARI); (iv) déterminer les proportions de cas confirmés de grippe chez les malades hospitalisés (SARI) et ceux atteints de syndromes grippaux (ILI) en consultation externe; (v) collecter des données qui seront utiles pour établir des estimations de la charge de morbidité en RDC.

Jusqu'à la fin de 2019, un total de 11 sites de surveillance sentinelle ont été opérationnels parmi lesquels 2 ambulatoires et 9 hospitaliers. Sur la base de la définition des cas, deux prélèvements oro et naso- pharyngés étaient effectués sur chaque patient et envoyés au laboratoire national de la grippe à l'INRB pour analyse au PCR-RT (Polymérase Chain Réactive -Real Time). Chaque échantillon était accompagné d'une fiche de notification reprenant les informations sociodémographiques et cliniques du patient. La présente étude visait à déterminer les profils épidémiologique, clinique et virologique des cas de grippe dans les 11 sites sentinelles qui étaient fonctionnels entre 2009 et 2018, avec comme objectifs spécifiques suivants: (i) décrire les caractéristiques sociodémographiques et cliniques des cas de grippe selon le sexe, l'âge, la catégorie et la provenance du malade, ainsi que la période de survenue des cas; (ii) déterminer les types et sous types des virus grippaux rencontrés ainsi que le schéma de leur circulation; (iii) déterminer l'ampleur des flambées épidémiques enregistrées par trimestre et par année en RDC.

## Méthodes

### Cadre de l'étude

Notre étude s'est focalisée sur les 11 sites de surveillance sentinelle qui étaient fonctionnels entre janvier 2009 et décembre 2018 et sur le laboratoire national de la grippe en RDC. Pour les sites sentinelles, il s'agissait à Kinshasa de l'hôpital général provincial de Kinshasa, de l'Hôpital Pédiatrique Kalembelembe, du centre médical RVA, du centre hospitalier Kingasani, du centre de santé Boyambi. Dans les provinces, il s'agissait de l'HGR Kinkanda à Matadi, du l'HGR Moanda, du HGR Kenya et de l'HGR Kisanga à Lubumbashi, du centre hospitalier Charité Maternelle à Goma, et de l'HGR Dipumba en Mbuji-Mayi. Le centre de santé Boyambi et le centre médical RVA étaient des sites ILI, tandis que les autres étaient à la fois ILI et SARI.

### Type d'étude

Il s'agissait d'une étude transversale faite moyennant une revue documentaire des fiches de notification des cas suspects envoyées au laboratoire nationale de la grippe et des registres des patients des sites sentinelles.

### Population de l'étude

La population de l'étude été constituée des patients qui avaient fréquentés les 11 sites sentinelles, entre le 1^er^ janvier 2009 et le 31 décembre 2018. Les patients étaient éligibles selon les critères suivants: avoir fréquenté un site sentinelle entre le 1^er^ janvier 2009 et le 31 décembre 2018, avoir répondu à la définition de cas ILI ou SARI, avoir subi des prélèvements naso et oro pharyngés et avoir été testé au virus influenza par la technique de PCR. Par contre, n'a pas été éligible à cette étude, tout patient ayant fréquenté une formation médicale qui n'est pas un site sentinelle, qui n'a pas répondu à la définition de cas ILI ou SARI, qui n'avait pas été prélevé et testé à la PCR.

### Technique d'échantillonnage

**Figure 1:**
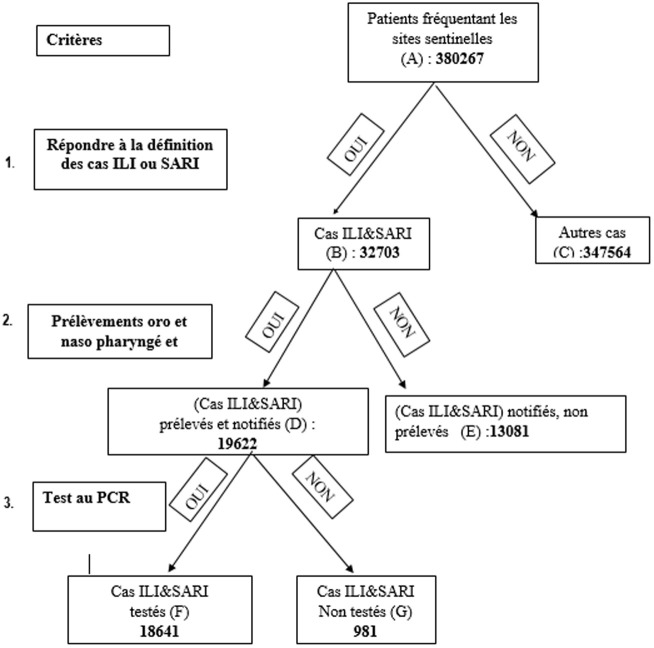
conception de l'étude

Nous avons procédé à un échantillonnage exhaustif constitué des patients qui ont répondu aux critères d'éligibilité. Le processus d'échantillonnage est repris à la Figure 1 qui représente la conception de l'étude. Cet échantillonnage a été préféré pour à la fois éviter le biais de sélection et augmenter la précision.

### Sources des données

Les données ont été collectées à partir de la base des données de fiches de notification envoyées au laboratoire national et de la base des données du système de surveillance. Elles ont été recueillies en décembre 2021 en utilisant le logiciel EPIINFO.7. Sur un total de 380867 patients qui avaient fréquentés les 11 sites sentinelles, 32708 cas ILI et SARI ont été enregistrés parmi lesquels, 19622 cas prélevés et notifiés, dont 18641 ont été testés à la PCR. Ces cas testés ont constitué la taille de notre échantillon.

### Traitement et analyse des données

Les variables telles que le sexe, l'âge, le site sentinelle de provenance, la catégorie du patient (ILI ou SARI), le type et sous-type viraux identifiés et la période de survenue des cas ont été traitées et analysées moyennant les logiciels Excel, Stata 14.0. Des méthodes statistiques descriptives et inférentielles ont été utilisées. Des fréquences, des pourcentages ainsi que des mesures d'association ou de risque telles que, p-value et le rapport de cotes (Odd Ratio) ont été calculées en utilisant l'analyse bivariée. Pour ce qui est de l'âge des patients comme variable quantitative, la médiane comme mesure de tendance centrale a été calculée. Les données manquantes ont été utilisées pour les statistiques descriptives, mais ignorées dans l'analyse bivariée.

**Considérations éthiques:** l'approbation par le comité d'éthique a été requise et obtenue.

## Résultats

Sur un total de 18461 échantillons analysés, les cas positifs étaient 1795 cas soit 9,7%.

### Analyse selon le sexe

Parmi les cas positifs, 1273 étaient ILI, soit 79%, et 522 SARI, soit 21%. Un total de 927 hommes étaient positifs, soit 51,6%, tandis que les femmes positives étaient 868, soit 48, 4%. En différentiant les cas ILI positifs de cas SARI positifs, les hommes représentaient respectivement 51,6% et 54% pour les cas ILI et SARI. Par contre, 49% des femmes étaient positives pour ILI contre 46% pour SARI. Ces différences n'ont pas étés statistiquement significatives avec une probabilité supérieure à 0,05 pour les deux situations, comme illustré au Tableau 1.

**Tableau 1:** caractéristiques sociodémographiques et cliniques des patients enrôlés

Type des patients	ILI (n=11659)				SARI (n=6802)			
Résultats d'analyse	Influenza positif	Influenza négatif	Valeur de p	OR 95%	influenza positif	influenza négatif	Valeur de p	OR à 95%
Effectif (%)	1273 (100)	10386 (100)			522 (100)	6280 (100)		
**Sexe**								
Masculin	647(51)	5065 (49)	0,17	1,1 (1-1,2)	280 (54)	3203 (51)	0,255	1,1(0,9-1,3)
Féminin	626(49)	5321 (51)		1	242 (46)	3077 (49)		1
**Age**								
Age médian	4 (0-84)	4 (0-98)			4 (0-86)	4 (0-97)		
< 2 ans	290 (23)	2743 (26)	0,055	1,7 (1-3,1)	270 (52)	3311(53)	0,290	1,4 (0,8-2,8)
2-4 ans	292 (23)	1950 (19)	0,000	2,4 (1,4-44)	117 (22)	844 (13)	0,003	2,4 (1,3-4,9)
5-14 ans	301 (24)	1257 (12)	0,000	3,1 (1,8-5,6)	52 (10)	425 (7)	0,023	2,1 (1,1-4,5)
15-39 ans	249 (19)	2472 (24)	0,104	1,6 (0,9-2,9)	36 (7)	840 (14)	0,361	0,47 (0,4-1,6)
40-64 ans	115 (9)	1681 (16)	0,451	1,1 (0,6-2,0)	32 (6)	638 (10)	0,720	0,89 (0,4-1,9)
≥ 65 ans	15 (1)	238 (2)		1	12 (2)	208 (3)		1
Inconnu	11 (1)	45 (0)			3 (1)	14 (0)		
**Symptômes**								
**Toux**								
Présent	1238 (97)	10137 (97)	0,376	1,6 (0,7-4,7)	515 (98)	6157 (98)	0,474	0,68 (0,2-3,6)
Absent	6 (1)	78 (1)		1	3 (1)	25 (0)		1
Inconnu	29 (2)	171 (2)			4 (1)	98 (2)		
**Fièvre**								
>38°C	1071 (84)	8667 (83)	0,000	1,6 (1,3-2,0)	484 (93)	5845 (93)	0,178	1,5 (0,8-2,8)
<38°C	164 (13)	1353 (13)		1	13 (5)	236 (4)		1
Inconnu	38 (3)	366 (4)			25 (2)	199 (3)		
**Mal de gorge**								
Présent	459 (36)	4947 (48)	0,000	0,7 (06-08)	296 (57)	3855 (61)	0,093	0,8 (0,7-1,0)
Absent	449 (35)	3277 (32)		1	135 (26)	1458 (23)		1
Inconnu	365 (29)	2162 (20)			91 (17)	967 (16)		
**Dyspnée**								
Présent	58 (5)	586 (6)	0,343	1,2 (0,9-1,6)	449 (86)	5612 (90)	0,68	0,9 (0,6-1,4)
Absent	820 (64)	7176 (69)		1	29 (6)	334 (5)		1
Inconnu	395 (31)	2624 (25)			44 (8)	334 (5)		
**Céphalées**								
Présent	304 (24)	2998 (29)	0,021	1,2 (1,02-1,4)	58 (11)	1074 (17)	0,011	1,6 (1,2-2,2)
Absent	620 (49)	5149 (50)		1	347 (67)	4024 (64)		1
Inconnu	349 (27)	2239 (21)			117 (22)	1182 (19)		
**Rhume**								
présent	1218 (96)	9843 (95)	0,055	1,5 (1-2,4)	489 (94)	5587 (89)	0,015	1,7 (11-33)
Absent	24 (2)	294 (3)		1	16 (3)	341 (5)		1
Inconnu	31 (2)	249 (2)			17 (3)	352 (6)		

### Analyse selon l'âge

L'âge médian des patients était de 4 ans (0-98). En considérant les tranches d'âge par rapport aux résultats positifs, la tranche d'âge < 2 ans occupait le premier rang avec un total de 560 cas soit 31,1% (n=1795) de cas positifs, tandis que la tranche d'âge ≥ 65 ans occupait le dernier rang avec 27 cas, soit1, 5% de cas positifs. Il y a eu 14 cas dont l'âge n'a pas été indiqué, soit 0,8%. Pris séparément entre ILI et SARI positifs, c'est la tranche d'âge de 5-14 ans qui occupait le premier rang avec 24% de cas ILI, tandis que pour les cas SARI positifs, c'est la tranche d'âge < 2 ans qui était première avec 52% comme cela est montré au Tableau 1. Ce tableau montre aussi que les enfants de moins de 5 ans représentaient un effectif total de 969, soit 54%. L'association entre l'âge et le résultat positif à l'influenza a été présente dans les tranches d'âge de 2-4 ans et de 5-14 ans, avec des valeurs p inférieures à 0,05.

### Analyse selon les symptômes des maladies

Dans cette étude, nous avons considéré les symptômes qui sont souvent rencontrés chez les patients ILI et SARI. Ce sont la fièvre, la toux, le mal de gorge, le rhume, les céphalées et la dyspnée. Les résultats repris dans le Tableau 1 nous montre que chez les patients ILI, la toux venait en première place avec 97%, suivi du rhume 96%, de la fièvre 84%, de la dyspnée 58%, du mal de gorge 36%, enfin des céphalées avec 24%. Pour les patients SARI, c'est toujours la toux qui était au premier rang avec 98%, suivi de la fièvre, 93%, de la dyspnée, 90% et de rhume avec 89%. Le mal de gorge et les céphalées étaient moins fréquents respectivement avec 64 et 17%. Chez les patients ILI comme SARI, il y avait une association entre la présence de la fièvre ≥ 38°C, de mal de gorge et l'absence de céphalées avec résultat positif à la grippe avec des valeurs p inférieures à 0.05. Par contre, pour les patients SARI, il y avait en plus la présence du rhume dans cette association.

### Analyse selon l'âge et le type et sous types viraux

En considérant la répartition des cas positifs (n=1795) selon les tranches d'âge et le type et sous type viraux, les résultats repris dans le Tableau 2 montrent qu'il y avait 1221 cas positifs au virus du type A, soit 68%, 566 cas positifs au virus du type B, soit 31,5%, et 8 cas de coinfection A&B. Parmi les 1221 cas positifs au virus du type A, le sous-type viral A(H3N2) comptait 595 cas, soit 46,6%, suivi du sous type A(HIN1)p (pandémique) avec 548 cas, soit 44,6%, enfin le sous type A(H1N1)s (saisonnier) avec 11 cas, soit 0,9%. Nous avons noté aussi 8 cas de coinfection A(H3N2) et A(H1N1)p, soit 0,3%, et 92 cas non sous typés, soit 7,5%. Ce tableau montre aussi qu'en calculant les pourcentages de chaque type par tranche d'âge, le type B avait respectivement 33,2% et 23,3% de cas dans les tranches d'âge < 2 ans et 2-4 ans, contre 30% et 22,6% pour le type A dans les mêmes tranches d'âge.

**Tableau 2:** répartition des cas positifs en selon les tranches d'âge et les types et sous-types viraux

	A(H1N1) p	A(H1N1) s	A(H3N2)	A H3N2&H1N1)	Non sous typé	Total Type A	% Type A	Type A&B	% Type A&B	Type B	% Type B	Total A, A&B, B
0<2 ans	161	5	172	2	31	**371**	30,4	1	12,5	188	33,2	**560**
2-4 ans	115	1	140	2	17	**275**	22,5	2	25,0	132	23,3	**409**
5-14 ans	117	1	94	0	19	**231**	18,9	3	37,5	119	21	**353**
15-39 ans	104	3	83	0	15	**205**	16,8	2	25,0	78	13,8	**285**
40-64 ans	40	1	63	0	6	**110**	9	0	0,0	37	6,5	**147**
≥ 65 ans	4	0	10	0	2	**16**	1,3	0	0,0	11	2	**27**
Inconnu	4	0	7	0	2	**13**	1,1	0	0,0	1	0,2	**14**
Total	545	11	569	4	92	**1221**	100	8	100,0	**566**	100,0	**1795**

### Analyse selon les sites de provenance et la catégorie des patients

**Figure 2:**
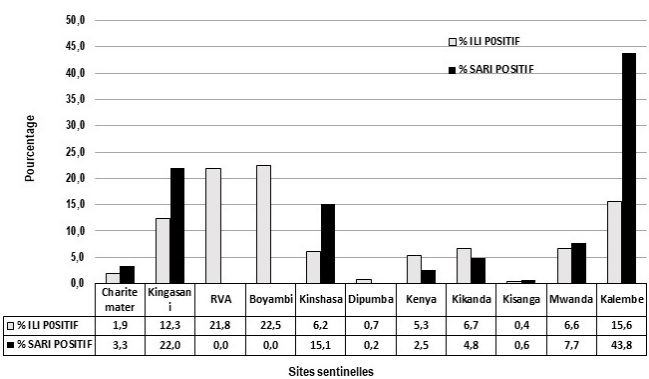
pourcentage des cas ILI et SARI positifs au virus influenza selon les sites sentinelles

Sur un total de 1795 cas positifs, Il y avait 1273 cas ILI soit, 71% et 522 cas SARI, soit 29%. En considérant les cas positifs ILI, le CS Boyambi était en premier rang avec 22,5%, suivi du CM RVA qui avait totalisé 21,8%. Quant aux cas positifs SARI, la première place revenait à HP Kalembelembe avec 43,8%, suivi du CH Kingasani qui avait 22,0%. Cette répartition des patients ILI et SARI selon les sites de provenance est reprise à la Figure 2.

### Analyse selon les trimestres et les années

Le Tableau 3 montre que le premier trimestre occupait le haut du classement avec 35,4% des cas, suivi du deuxième trimestre qui avait totalisé 30,4%, ensuite venait le quatrième trimestre avec 29,8%. Le troisième trimestre se plaçait en dernière position avec 4,5% des cas. Par rapport à la circulation des souches virales, on observe que le sous type A(H3N2) était plus fréquent au premier trimestre de l'année, le type B au deuxième et troisième trimestre et le sous type A(H1N1)p était plus fréquent au quatrième trimestre. Pour ce qui est de la répartition annuelle des cas, la taille des effectifs variait entre 32 et 271, soit une médiane de 232 (32-271). Cette répartition reprise au Tableau 3 montre que l'année 2009 occupait le premier rang avec 15,1%, suivi de l'année 2013 avec 14%, ensuite vient l'année 2014 avec 13,6%, et enfin l'année 2018 avec 1,8%. Par rapport au virus du type A, on observe que le sous type A (H1N1)p était la souche la plus fréquente en 2009, 2012, 2015, 2016 et 2017. Par contre la souche H3N2 était prédominante en 2011, 2014 et 2018, tandis que le type B était plus fréquent en 2010, 2013 et 2018. A partir de 2010, la souche A(H1N1)s saisonnier avait disparu. Par rapport à la circulation des souches virales prédominantes, on observe l'alternance selon les années sous forme d'un cycle “A(H1N1)p-B-A(H3N2)-A(H1N1)p”.

**Tableau 3:** répartition de cas positifs par trimestre et par année selon les types et les sous-types du virus grippal

Période	A (H1N1)p	A (H1N1)s	A(H3N2)	H3N2 & A (H1N1)p	Non sous typé	Type A&B	Type B	Total positifs	% positifs
**Trimestre**									
Trimestre 1	114	10	313	0	1	29	168	635	35,4
Trimestre 2	101	1	117	1	5	19	301	545	30,4
Trimestre 3	17	0	24	0	1	3	35	80	4,5
Trimestre 4	313	0	115	3	1	41	62	535	29,8
**Total trimestre**	**545**	**11**	**569**	**4**	**8**	**92**	**566**	**1795**	**100**
**Année**									
2009	176*	11	39	3	24	0	18	271	15,1
2010	11	0	77	0	2	3	148*	241	13,4
2011	40	0	138*	0	19	1	38	236	13,1
2012	67*	0	31	0	5	1	11	115	6,4
2013	35	0	101	0	8	1	107*	252	14
2014	12	0	105*	0	27	0	101	245	13,6
2015	110*	0	42	1	4	2	69	228	12,7
2016	45*	0	15	0	3	0	35	98	5,5
2017	43*	0	11	0	0	0	23	77	4,3
2018	6	0	10	0	0	0	16*	32	1,8
**Total années**	**545**	**11**	**569**	**4**	**92**	**8**	**566**	**1795**	**100**

* Souche virale prédominante au cours de l’année

## Discussion

### Enoncé des principaux résultats

Les résultats de notre étude ont montré que la grippe représentait 9,8% parmi les autres infections respiratoires aiguës sévères; les enfants de moins de 5 ans étaient les plus touchés par la grippe dans la capitale comme dans les provinces de la RDC; les symptômes comme la fièvre ≥ 38°C, le mal de gorge, les céphalées, étaient associés à l'infection grippales; les souches virales A(H3N2), A(H1N1)p et B circulaient concomitamment chaque année, mais à des proportion différentes; le schéma de circulation était “A(H1N1)-B-A(H3N2)-A(H1N1)p”; les flambées grippales s'observaient plus aux premiers, deuxième et quatrième trimestres de l'année.

### Forces et faiblesses de l'étude

Comme force de notre étude, nous citerons: la prise en compte d'aspects épidémiologiques, cliniques et virologiques, une importante taille de l'échantillon de l'ordre de 18641 patients enrôlés, l'approbation du comité d'éthique pour sa mise en œuvre. Comme faiblesse, il y a le fait que l'évolution clinique des patients en termes de guérison ou de décès n'a pas été élucidée. En plus, il n'y a pas eu de sites sentinelles situés dans des milieux ruraux très reculés.

**Forces et faiblesses par rapport aux autres études:** par rapport aux autres études, les forces sont: la période couverte qui est suffisamment longue (dix ans) et le recours à des évidences comme les résultats des analyses de laboratoire. Nous citerons comme faiblesse, l'absence de données plus récentes.

### Discussion des différences importantes dans les résultats

Selon les résultats de notre étude, les cas positifs au virus grippal représentaient respectivement 51,6% pour les hommes et 48,4% pour les femmes. Cette différence n'est pas statistiquement significative, p>0,05. Ce résultat est différent de ceux trouvés dans certaines études pour lesquels le sexe féminin constituait un facteur de risque [9-11], mais similaires à ceux trouvés dans nombre des pays africains [5,6].

Par rapport à l'âge, les résultats de notre étude ont montré que les tranches d'âge de < 2 ans et 2-4 ans représentaient environ 66% de cas positifs SARI et 46% de cas positifs ILI, d'où l'intérêt d'orienter des interventions prioritaires en matière de lutte contre la grippe à ce groupe cible en l'occurrence la vaccination et la prise en charge des cas. Katz *et al*. en 2009 ont aussi trouvé au Kenya, sur une étude portant sur les résultats de 6 premières années de la surveillance sentinelle de la grippe, que c'est la tranche d'âge de moins de 5 ans et spécifiquement celle en dessous de 2 ans, qui était la plus affectée, avec 33% de cas [12]. Plusieurs autres études à travers l'Afrique ont montré le lourd fardeau que portent les enfants de moins de 5 ans en termes de morbidité et de mortalité dues à la grippe [3-8,13,14]. En outre, l'absence de données sur le suivi des complications et de la mortalité par tranche d'âge sur les fiches de notification ne nous a pas permis de déterminer le groupe d'âge à risque des complications et de décès. Concernant la répartition des cas confirmés positifs selon les tranches d'âge et le type et sous-type viraux, les résultats de notre études ont montré qu'il y avait plus de cas positifs du sous-type viral A(H3N2) dans les tranche d'âge < 2 ans, 2-4 ans, et au-delà de 40 ans que de cas positifs au sous-type viral virus A(H1N1)p. Par contre, le sous type A(H1N1)p était prédominant dans les tranches d'âge de 5-14 et 15-39 ans.

En Zambie, Theo *et al*. en 2009 ont eu les mêmes résultats que ceux de notre étude à propos des enfants âgés de moins de 5 ans qui étaient plus touchés par le sous type viral A(H3N2) et le type B. Néanmoins, la différence se trouve dans les tranches touchées par le A(H1N1)p qui en Zambie [8] concernait les personnes âgé de 5 à 24 ans, alors que dans notre étude, les personnes infectées étaient comprises entre 5 et 39 ans. D'autre études, comme celle de Dalhatu *et al*., en 2010 au Nigeria [6] et celle de Barakat *et al*. en 2009 au Bangladesh [15] ont montré que le sous type A(H1N1)p n'était pas fréquent en dessous de 5 ans. Les résultats obtenus par ces deux chercheurs diffèrent sur l'intervalle d'âge des personnes touchées par ce sous-type. Le premier a trouvé que le sous-type A(H1N1)p était fréquent entre 5 et 17 ans et au-delà de 65 ans, le second a trouvé plutôt l'intervalle entre 5 et 55 ans. La différence des résultats entre chercheurs peut s'expliquer entre autres par la différence de tranches d'âge utilisées par chacun. Par rapport aux symptômes manifestés par les patients, la toux n'était pas associée à l'infection au virus influenza, alors qu'elle est considérée comme un des symptômes majeurs de cette infection. Ce résultat est différent de ceux trouvés dans nombreuses études [16-18]. Par ailleurs, les céphalées étaient associées à l'infection grippale. Cette situation est similaire à celle rapportée par d'autres chercheurs [16,18].

Concernant la répartition des cas positifs selon la catégorie des patients et leurs sites de provenance, notre étude a trouvé que les cas ILI représentaient 79% et les cas SARI, 21%. Cette différence pourrait s'expliquer par trois suppositions. La première est l'existence de deux sites sentinelles qui ne collectaient que des cas ILI (CM RVA et CH Boyambi); la deuxième serait la faiblesse de la sensibilité de la définition des cas SARI par rapport aux cas ILI [19]; et la troisième qui découle de la deuxième serait l'arrivée souvent tardive des cas SARI à l'hôpital au-delà du délai requis pour le prélèvement. Pour ce qui est de la provenance des patients, HP Kalembelembe a enregistré à lui seul 43,8% des cas SARI. Nous pensons que ce résultat se justifie dans la mesure où ce site est un hôpital pédiatrique de référence du niveau provincial, et par conséquent accueille des cas graves y compris le cas SARI. Par rapport au trimestre de l'année, notre étude a trouvé que le troisième trimestre (juillet, aout et septembre) avait moins des cas positifs au virus influenza. Cette période pourrait être propice pour la vaccination des masses. Cela pourrait s'expliquer par le fait que ce trimestre coïncide avec la saison sèche, Par contre les autres trimestres avaient des flambées épidémiques qui coïncident avec la saison de pluie. D'autres pays à climat tropical en Afrique et en Asie ont connu des flambées de grippe pendant la saison de pluie. C'est le cas du Kenya, du Rwanda, de la Thaïlande, de Singapour, du Bangladesh... [20-26].

Par rapport à la circulation des types et sous-types viraux, nous avons constaté que le sous type A (H1N1)p était la souche la plus fréquente en 2009, 2012, 2015, 2016 et 2017. Par contre la souche H3N2 était prédominante en 2011, 2014 et 2018, tandis que le type B était plus fréquent en 2010, 2013 et 2018. A l'opposé de la RDC qui avait une prédominance du virus A(H1N1)p en 2009, l'Afrique de l'ouest était sous l'emprise du virus A(H3N2) à la même période. Au moment où le virus du type B était prédominant en RDC et l'Afrique australe en 2010, l'Afrique de l'Ouest était principalement touchée par le sous type A(H1N1)p [27]. Cette situation montre que les virus grippaux circulent différemment d'une région à l'autre. Certaines études ont incriminé la baisse de température ou une forte humidité comme des facteurs de transmission [28-32]. Ces facteurs diffèrent selon les zones géographiques.

Les résultats de notre étude ont montré un schéma de circulation virale sous forme d'une boucle dans l'ordre suivant A(H1N1)-B-A(H3N2)-A(H1N1)p. Au Cameroun dans une étude menée par Njoum en 2014, on retrouve le même schéma entre 2009 et 2013 [33]. Ce schéma de circulation constituerait une hypothèse de recherche pouvant cibler plusieurs pays. Par ailleurs, nous avons noté une diminution de cas rapportés en 2016, 2017 et 2018. Cette situation est due à la diminution drastique du financement du système de surveillance de l'ordre de 80%. Notre étude a montré que l'année 2009 a eu plus de cas que les autres années. Ce fait est dû à la survenue de la pandémie au virus A(H1N1)p en 2009.

### Sens de l'étude

Le sens de notre étude était d'aborder les caractéristiques épidémiologiques, cliniques et virologiques de personnes touchées par la grippe saisonnière ayant fréquentées les sites sentinelles de surveillance de 2009 à 2018. Pour ce faire, nous devrions répondre aux questions suivantes: Qui étaient ces personnes touchées par la grippe? Quelles étaient les manifestations cliniques qu'elles présentaient? De quels lieux provenaient-elles? Par quels types et sous-types étaient-elles infectées?

**Les questions sans réponse et les recherches futures:** la question de recherche future est de vérifier si le schéma de circulation observé ...A(H1N1)p-B-A(H3N2)-A(H1N1)p... est présent dans d'autres régions d'Afrique ou d'ailleurs.

**Généralisation des résultats:** malgré certaines limites que nous avons évoquées, nous pensons que ces résultats sont valables et ont une portée nationale.

## Conclusion

L'infection humaine à virus influenza appelée grippe, représente 9,8% des infections respiratoires aiguës en RDC, sévit pendant la saison de pluie et touche le plus souvent les enfants, particulièrement ceux de moins de 5 ans, avec une prédominance des cas SARI par rapport aux cas ILI. Cette étude a aussi montré que les sous-types A(H1N1)p, H3N2 et le type B étaient en circulation d'une manière concomitante. Selon les souches dominantes annuelles, il y aurait une alternance de l'ordre de “ A(H1N1)p-B-A(H3N2)-A(H1N1)p”. La disparition de la souche A(H1N1)s (saisonnière), au profit de la souche A (H1N1)p (pandémique) qui est une souche triplement assortie (homme, volaille, porc), prouve à suffisance que la grippe demeure une menace pour la santé publique contre laquelle le système de surveillance sentinelle devrait être renforcé et intégré au système de surveillance animale dans le cadre d'une seule santé.

### Etat des connaissances sur le sujet

- Les enfants de moins de 5 ans sont les plus touchés par la grippe;
- Les souches en circulation en RDC sont A(H1N1)p, H3N2 et le type B.

### Contribution de notre étude à la connaissance

- Dans la circulation virale, il y aurait une alternance selon les souches dominantes de l'ordre “A(H1N1)p-B-A(H3N2)-A(H1N1)p”;
- La toux prise isolément n'est pas associée à la grippe.

## Remerciements

Nous tenons à remercier le personnel des sites sentinelles de surveillance la grippe et le personnel du laboratoire national de la grippe pour avoir généré les données primaires.
